# Supplementary material for: Tuning the 0 − π Josephson junction with a magnetic impurity: Role of tunnel contacts, exchange coupling, e − e interactions and high-spin states
Source: Sci Rep. 2018 Mar 26;8:5208. doi: 10.1038/s41598-018-23517-w (PMC5979961; doi:10.1038/s41598-018-23517-w)
Supplement: Supplementary file 1 — Supplementary Material [file 41598_2018_23517_MOESM1_ESM.pdf]

Supplementary Information

Tuning the $0-\pi$ Josephson junction with a magnetic impurity: Role of tunnel contacts, exchange coupling, $e-e$ interactions and high-spin states

Subhajit Pal and Colin Benjamin*

School of Physical Sciences, National Institute of Science Education & Research, HBNI, Jatni-752050, India

In section I we first introduce our model, provide a theoretical background to our study with wavefunctions and boundary conditions to calculate the Josephson current. In section II we give the explicit form of 8×8 matrix M. The explicit form of Andreev bound states is given in section III. Finally, in section IV we provide spin flip probability (F_2) values of the high spin magnetic impurity (HSM) for different values of impurity spin (S) and magnetic moment (m') in a tabular format.

I. WAVEFUNCTIONS AND BOUNDARY CONDITIONS IN THE JOSEPHSON JUNCTION IN PRESENCE OF A HIGH SPIN MAGNETIC IMPURITY

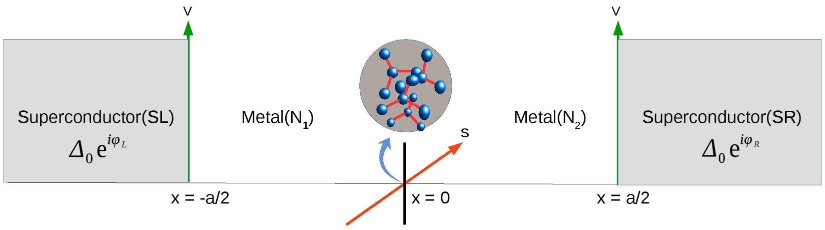

FIG. 1: Josephson junction composed of two normal metals and a high spin magnetic impurity with spin S and magnetic moment m' at $x = 0$ sandwiched between two s-wave superconductors.

We consider a system consists of two normal metals with a HSM sandwiched between two conventional s-wave singlet superconductors. Our model is shown in Fig. 1, it depicts a HSM at $x = 0$ and two superconductors at $x < -a/2$ and $x > a/2$. There are normal metal regions in $-a/2 < x < 0$ and $0 < x < a/2$.

A. Wavefunctions

There can be eight different types of quasiparticle injection into our system: an electron-like quasiparticle (ELQ) with spin up or down or a hole-like quasiparticle (HLQ) with spin up or down injected from either the left or from the right superconducting electrode. For the injection of spin up electron in left superconductor, the wave function is given by[1]-

$$\psi_{SL}(x) = \begin{pmatrix} u \\ 0 \\ 0 \\ v \end{pmatrix} e^{iq_+x}\phi^S_{m'} + r^{\uparrow\uparrow}_{ee} \begin{pmatrix} u \\ 0 \\ 0 \\ v \end{pmatrix} e^{-iq_+x}\phi^S_{m'} + r^{\uparrow\downarrow}_{ee} \begin{pmatrix} 0 \\ u \\ -v \\ 0 \end{pmatrix} e^{-iq_+x}\phi^S_{m'+1} + r^{\uparrow\uparrow}_{eh} \begin{pmatrix} 0 \\ -v \\ u \\ 0 \end{pmatrix} e^{iq_-x}\phi^S_{m'+1} + r^{\uparrow\downarrow}_{eh} \begin{pmatrix} v \\ 0 \\ 0 \\ u \end{pmatrix} e^{iq_-x}\phi^S_{m'}, \quad \text{for } x < -\frac{a}{2}$$

The amplitudes $r^{\uparrow\uparrow}_{ee}, r^{\uparrow\downarrow}_{ee}, r^{\uparrow\uparrow}_{eh}, r^{\uparrow\downarrow}_{eh}$ represent normal reflection, normal reflection with spin flip, Andreev reflection with spin flip and Andreev reflection without flip respectively.

The corresponding wave function in the right superconductor is-

$$\psi_{SR}(x) = t_{ee}^{\uparrow\uparrow}\begin{pmatrix} ue^{i\varphi} \\ 0 \\ 0 \\ v \end{pmatrix} e^{iq_+x}\phi^S_{m'} + t_{ee}^{\uparrow\downarrow}\begin{pmatrix} 0 \\ ue^{i\varphi} \\ -v \\ 0 \end{pmatrix} e^{iq_+x}\phi^S_{m'+1} + t_{eh}^{\uparrow\uparrow}\begin{pmatrix} 0 \\ -ve^{i\varphi} \\ u \\ 0 \end{pmatrix} e^{-iq_-x}\phi^S_{m'+1} + t_{eh}^{\uparrow\downarrow}\begin{pmatrix} ve^{i\varphi} \\ 0 \\ 0 \\ u \end{pmatrix} e^{-iq_-x}\phi^S_{m'}, \text{for } x > \frac{a}{2}$$

where $t_{ee}^{\uparrow\uparrow}, t_{ee}^{\uparrow\downarrow}, t_{eh}^{\uparrow\uparrow}, t_{eh}^{\uparrow\downarrow}$ are the transmission amplitudes, corresponding to the reflection process described above and $\varphi = \varphi_R - \varphi_L$ is the phase difference between right side and left side superconductor. $\phi^S_{m'}$ is the eigenspinor of the HSM, with its S^z operator acting as- $S^z\phi^S_{m'} = m'\phi^S_{m'}$, with m' being the spin magnetic moment of the HSM. The BCS coherence factors are defined as $u = \sqrt{\frac{1}{2}\left(1 + \frac{\sqrt{E^2-\Delta_0^2}}{E}\right)}$, $v = \sqrt{\frac{1}{2}\left(1 - \frac{\sqrt{E^2-\Delta_0^2}}{E}\right)}$. $q_\pm = \sqrt{\frac{2m^\star}{\hbar^2}(E_F \pm \sqrt{E^2 - \Delta_0^2})}$ is the wavevector for electron-like quasiparticle (q_+) and hole-like quasiparticle (q_-) in the left and right superconducting wavefunctions, ψ_{SL} and ψ_{SR}. The wavefunction in the normal metal region (N_1) is given by-

$$\psi_{N_1}(x) = (ee^{ik_e(x+a/2)} + fe^{-ik_ex})\begin{pmatrix} 1 \\ 0 \\ 0 \\ 0 \end{pmatrix}\phi^S_{m'} + (e'e^{ik_e(x+a/2)} + f'e^{-ik_ex})\begin{pmatrix} 0 \\ 1 \\ 0 \\ 0 \end{pmatrix}\phi^S_{m'+1}$$

$$+(ge^{-ik_h(x+a/2)} + he^{ik_hx})\begin{pmatrix} 0 \\ 0 \\ 1 \\ 0 \end{pmatrix}\phi^S_{m'+1} + (g'e^{-ik_h(x+a/2)} + h'e^{ik_hx})\begin{pmatrix} 0 \\ 0 \\ 0 \\ 1 \end{pmatrix}\phi^S_{m'}, \text{for } -\frac{a}{2} < x < 0$$

Similarly the wavefunction in the normal metal region (N_2) is given by-

$$\psi_{N_2}(x) = (a_0e^{ik_ex} + be^{-ik_e(x-a/2)})\begin{pmatrix} 1 \\ 0 \\ 0 \\ 0 \end{pmatrix}\phi^S_{m'} + (a'e^{ik_ex} + b'e^{-ik_e(x-a/2)})\begin{pmatrix} 0 \\ 1 \\ 0 \\ 0 \end{pmatrix}\phi^S_{m'+1}$$

$$+(ce^{-ik_hx} + de^{ik_h(x-a/2)})\begin{pmatrix} 0 \\ 0 \\ 1 \\ 0 \end{pmatrix}\phi^S_{m'+1} + (c'e^{-ik_hx} + d'e^{ik_h(x-a/2)})\begin{pmatrix} 0 \\ 0 \\ 0 \\ 1 \end{pmatrix}\phi^S_{m'}, \text{for } 0 < x < \frac{a}{2}$$

$k_{e,h} = \sqrt{\frac{2m^\star}{\hbar^2}(E_F \pm E)}$ is the wave vector in the normal metals. In our work we have used the Andreev approximation[2] $q_+ = q_- = k_F$ and $k_{e,h} \approx k_F \pm \frac{k_FE}{2E_F}$, where k_F is the Fermi wavevector, with $E_F >> \Delta$.

B. Boundary conditions

The boundary conditions at $x = -a/2$:

$$\psi_{SL}(x) = \psi_{N_1}(x), \text{(continuity of wavefunctions)}$$

$$\frac{d\psi_{N_1}}{dx} - \frac{d\psi_{SL}}{dx} = \frac{2m^\star V}{\hbar^2}\psi_{N_1}, \text{(discontinuity in first derivative)}$$

and at $x = 0$:

$$\psi_{N_1}(x) = \psi_{N_2}(x)$$

$$\frac{d\psi_{N_2}}{dx} - \frac{d\psi_{N_1}}{dx} = -\frac{2m^\star J_0\vec{s}.\vec{S}}{\hbar^2}\psi_{N_1}$$

where $\vec{s}.\vec{S}$ is the exchange operator in the Hamiltonian and is given by $\vec{s}.\vec{S} = s^z S^z + \frac{1}{2}(s^- S^+ + s^+ S^-)$;

$$\vec{s}.\vec{S}\begin{pmatrix}1\\0\\0\\0\end{pmatrix}\phi^S_{m'} = mm'\begin{pmatrix}1\\0\\0\\0\end{pmatrix}\phi^S_{m'} + \frac{1}{2}F_1F_2\begin{pmatrix}0\\1\\0\\0\end{pmatrix}\phi^S_{m'+1}$$

and

$$\vec{s}.\vec{S}\begin{pmatrix}0\\1\\0\\0\end{pmatrix}\phi^S_{m'+1} = (m-1)(m'+1)\begin{pmatrix}0\\1\\0\\0\end{pmatrix}\phi^S_{m'+1} + \frac{1}{2}F_1F_2\begin{pmatrix}1\\0\\0\\0\end{pmatrix}\phi^S_{m'}$$

Here $F_1 = \sqrt{(s+m)(s-m+1)}$ is the spin-flip probability for electron, $F_2 = \sqrt{(S-m')(S+m'+1)}$ is the spin-flip probability[3] for HSM, m is the spin magnetic moment of the spin up electron ($m = 1/2$) and $m-1$ is the spin magnetic moment of the spin down electron ($m-1 = -1/2$). $s^\pm = s_x \pm is_y$ and $S^\pm = S_x \pm iS_y$ are the raising and lowering spin operators.
Finally, at $x = a/2$:

$$\psi_{N_2}(x) = \psi_{SR}(x)$$

$$\frac{d\psi_{SR}}{dx} - \frac{d\psi_{N_2}}{dx} = \frac{2m^\star V}{\hbar^2}\psi_{N_2}.$$

We will later use the dimensionless parameter $J = \frac{m^\star J_0}{\hbar^2 k_F}$ as a measure of strength of exchange interaction and $Z = \frac{V}{V_0}$, with $V_0 = \frac{\hbar^2 k_F}{m^*}$ as a measure of interface transparency. Thus a value of $Z = 5$ (say) means interface potential $V = 5V_0$, with V in units of V_0. By using above boundary conditions one can get the different scattering amplitudes. The wave functions for the other seven types of quasiparticle injection process are constructed in the same way.

II. EXPLICIT FORM OF MATRIX M

To calculate the bound state contribution of total Josephson current we introduce a 8×8 matrix M in Eq. (4) in section 3.2 of our paper which is given by-

$$M = \begin{pmatrix}M_{11}&M_{12}&M_{13}&M_{14}&M_{15}&M_{16}&M_{17}&M_{18}\\M_{21}&M_{22}&M_{23}&M_{24}&M_{25}&M_{26}&M_{27}&M_{28}\\M_{31}&M_{32}&M_{33}&M_{34}&M_{35}&M_{36}&M_{37}&M_{38}\\M_{41}&M_{42}&M_{43}&M_{44}&M_{45}&M_{46}&M_{47}&M_{48}\\M_{51}&M_{52}&M_{53}&M_{54}&M_{55}&M_{56}&M_{57}&M_{58}\\M_{61}&M_{62}&M_{63}&M_{64}&M_{65}&M_{66}&M_{67}&M_{68}\\M_{71}&M_{72}&M_{73}&M_{74}&M_{75}&M_{76}&M_{77}&M_{78}\\M_{81}&M_{82}&M_{83}&M_{84}&M_{85}&M_{86}&M_{87}&M_{88}\end{pmatrix}$$

where,

$$M_{11} = (-ie^{ik_Fa}u + e^{ik_Fa}uZ - e^{2ik_Fa}uZ)$$
$$M_{12} = 0$$
$$M_{13} = 0$$
$$M_{14} = -v(ie^{ik_Fa} - Z + e^{ik_Fa}Z)$$
$$M_{15} = (ie^{ik_Fa+i\varphi}u - e^{ik_Fa+i\varphi}uZ + e^{2ik_Fa+i\varphi}uZ)$$
$$M_{16} = 0$$
$$M_{17} = 0$$
$$M_{18} = (ie^{ik_Fa+i\varphi}v + e^{ik_Fa+i\varphi}vZ - e^{i\varphi}vZ)$$
$$M_{21} = 0$$
$$M_{22} = (ie^{ik_Fa}u - e^{ik_Fa}uZ + e^{2ik_Fa}uZ)$$
$$M_{23} = -v(ie^{ik_Fa} - Z + e^{ik_Fa}Z)$$
$$M_{24} = 0$$
$$M_{25} = 0$$
$$M_{26} = (-ie^{ik_Fa+i\varphi}u + e^{ik_Fa+i\varphi}uZ - e^{2ik_Fa+i\varphi}uZ)$$
$$M_{27} = (ie^{ik_Fa+i\varphi}v + e^{ik_Fa+i\varphi}vZ - e^{i\varphi}vZ)$$
$$M_{28} = 0$$
$$M_{31} = 0$$
$$M_{32} = (ie^{ik_Fa}v - e^{ik_Fa}vZ + e^{2ik_Fa}vZ)$$
$$M_{33} = -u(ie^{ik_Fa} - Z + e^{ik_Fa}Z)$$
$$M_{34} = 0$$
$$M_{35} = 0$$
$$M_{36} = (-ie^{ik_Fa}v + e^{ik_Fa}vZ - e^{2ik_Fa}vZ)$$
$$M_{37} = (ie^{ik_Fa}u - uZ + e^{ik_Fa}uZ)$$
$$M_{38} = 0$$
$$M_{41} = (-ie^{ik_Fa}v + e^{ik_Fa}vZ - e^{2ik_Fa}vZ)$$
$$M_{42} = 0$$
$$M_{43} = 0$$
$$M_{44} = -u(ie^{ik_Fa} - Z + e^{ik_Fa}Z)$$
$$M_{45} = (ie^{ik_Fa}v - e^{ik_Fa}vZ + e^{2ik_Fa}vZ)$$
$$M_{46} = 0$$
$$M_{47} = 0$$
$$M_{48} = (ie^{ik_Fa}u - uZ + e^{ik_Fa}uZ)$$
$$M_{51} = (2e^{2ik_Fa}Jmm'u(i - 2Z) + 2e^{3ik_Fa}(-i + Jmm')uZ + 2e^{ik_Fa}(i + Jmm')u(-i + Z))$$
$$M_{52} = (e^{2ik_Fa}F_1F_2Ju(i - 2Z) + e^{3ik_Fa}F_1F_2JuZ + e^{ik_Fa}F_1F_2Ju(-i + Z))$$
$$M_{53} = (-F_1F_2JvZ - e^{2ik_Fa}F_1F_2Jv(i + Z) + e^{ik_Fa}F_1F_2Jv(i + 2Z))$$
$$M_{54} = (2e^{2ik_Fa}v(1 - iZ) + 2(i + Jmm')vZ + 2e^{2ik_Fa}Jmm'v(i + Z) - 2e^{ik_Fa}Jmm'v(i + 2Z))$$
$$M_{55} = -2e^{2ik_Fa+i\varphi}u$$
$$M_{56} = 0$$
$$M_{57} = 0$$
$$M_{58} = -2e^{i(k_Fa+\varphi)}v$$
$$M_{61} = (e^{2ik_Fa}F_1F_2Ju(i - 2Z) + e^{3ik_Fa}F_1F_2JuZ + e^{ik_Fa}F_1F_2Ju(-i + Z))$$
$$M_{62} = 2e^{ik_Fa}u(1 + (-1 + e^{ik_Fa})J(-1 + m)(1 + m')(i + (-1 + e^{ik_Fa})Z) + 2e^{ik_Fa}Z\sin(k_Fa))$$

$M_{63} = 2v((-i - J(-1+m)(1+m'))Z - e^{2ik_Fa}(-i + J(-1+m)(1+m'))(i+Z) + e^{ik_Fa}J(-1+m)(1+m')(i+2Z))$

$M_{64} = (F_1F_2JvZ + e^{2ik_Fa}F_1F_2Jv(i+Z) - e^{ik_Fa}F_1F_2Jv(i+2Z))$

$M_{65} = 0$

$M_{66} = -2e^{ik_Fa+i(k_Fa+\varphi)}u$

$M_{67} = 2e^{i(k_Fa+\varphi)}v$

$M_{68} = 0$

$M_{71} = e^{-ik_Fa}(e^{2ik_Fa}F_1F_2Jv(i-2Z) + e^{3ik_Fa}F_1F_2JvZ + e^{ik_Fa}F_1F_2Jv(-i+Z))$

$M_{72} = 2v(-(-1+e^{ik_Fa})J(-1+m)(1+m')(i+(-1+e^{ik_Fa})Z) + i(i+(-1+e^{2ik_Fa})Z))$

$M_{73} = e^{-ik_Fa}(2(i+J(m-1)(m'+1))uZ + 2e^{2ik_Fa}(-i+J(m-1)(m'+1))u(i+Z) - 2e^{ik_Fa}J(m-1)(m'+1)u(i+2Z))$

$M_{74} = e^{-ik_Fa}(F_1F_2JuZ + e^{2ik_Fa}F_1F_2Ju(i+Z) - e^{ik_Fa}F_1F_2Ju(i+2Z))$

$M_{75} = 0$

$M_{76} = 2e^{ik_Fa}v$

$M_{77} = -2u$

$M_{78} = 0$

$M_{81} = e^{-ik_Fa}(2e^{ik_Fa}v(1+iZ) + 2e^{3ik_Fa}(-i+Jmm')vZ + 2e^{ik_Fa}Jmm'v(-i+Z) - 2e^{2ik_Fa}Jmm'v(-i+2Z))$

$M_{82} = e^{-ik_Fa}(-e^{2ik_Fa}F_1F_2Jv(i-2Z) - e^{3ik_Fa}F_1F_2JvZ - e^{ik_Fa}F_1F_2Jv(-i+Z))$

$M_{83} = e^{-ik_Fa}(F_1F_2JuZ + e^{2ik_Fa}F_1F_2Ju(i+Z) - e^{ik_Fa}F_1F_2Ju(i+2Z))$

$M_{84} = e^{-ik_Fa}(2(i+Jmm')uZ + 2e^{2ik_Fa}(-i+Jmm')u(i+Z) - 2e^{ik_Fa}Jmm'u(i+2Z))$

$M_{85} = -2e^{ik_Fa}v$

$M_{86} = 0$

$M_{87} = 0$

$M_{88} = -2u$

III. EXPLICIT FORM OF ANDREEV BOUND STATES

Andreev bound states are described by Eq. 5 (section 3.2) of our manuscript, herein below we explicitly describe the various terms $A(\varphi)$, $B(\varphi)$ and C occuring in the aforesaid equation.

$$
\begin{aligned}
A(\varphi) = & -2e^{3ik_Fa}((1+2Z^2)(8(1+2Z^2)^2+J^4(F_2^2+m'+m'^2)^2(1+10(Z^2+Z^4))+J^2(3+6m'(1+m')+8Z^2\\
&+2(F_2^2-8(F_2^2+m'+m'^2)Z^2-4(-1+2F_2^2+2m'(1+m'))Z^4)))+2Z^2(1+2Z^2)(16+16J^3(F_2^2+m'+m'^2)Z\\
&-16Z^2+3J^4(F_2^2+m'+m'^2)^2(-1+Z^2)+4J^2(-1+2F_2^2+2m'(1+m'))(-1+Z^2))\cos(2k_Fa)-2Z^3(16Z(-3+Z^2)\\
&+J^4(F_2^2+m'+m'^2)^2Z(-3+Z^2)+4J^2(-1+2F_2^2+2m'(1+m'))Z(-3+Z^2)+16J(-1+3Z^2)+4J^3(F_2^2+m'\\
&+m'^2)(-1+3Z^2))\cos(3k_Fa)+8\cos(\varphi)+(16Z^2-J^2(-1+2F_2^2-2m'(1+m'))(1+2Z^2))\cos(\varphi)+2Z\cos(k_Fa)(8Z\\
&+12J(1+2Z^2)^2+J^2Z(-3-2F_2^2-6m'(1+m')-4Z^2+8(F_2^2+m'+m'^2)Z^2+4(-1+2F_2^2+2m'(1+m'))Z^4)\\
&+16(Z^3+Z^5)-4J^3(F_2^2+m'+m'^2)(1+5(Z^2+Z^4))-3J^4(F_2^2+m'+m'^2)^2Z(1+5(Z^2+Z^4))+(8Z+J(4\\
&+J(-1+2F_2^2-2m'(1+m'))Z))\cos(\varphi))+2Z(-8+12JZ(1+2Z^2)^2-16(Z^2+Z^4)+J^2(3+2F_2^2+6m'(1+m')\\
&+4Z^2-8(F_2^2+m'+m'^2)Z^2-4(-1+2F_2^2+2m'(1+m'))Z^4)+3J^4(F_2^2+m'+m'^2)^2(1+5(Z^2+Z^4))\\
&-4J^3(F_2^2+m'+m'^2)Z(1+5(Z^2+Z^4))+(-8+J^2(1-2F_2^2+2m'(1+m'))+4JZ)\cos(\varphi))\sin(k_Fa)\\
&-4Z^2(1+2Z^2)(-16Z+J^2(-4Z+(F_2^2+m'+m'^2)(8Z+J(4+3J(F_2^2+m'+m'^2)Z-4Z^2))))\sin(2k_Fa)\\
&-2Z^3(16-48Z^2+16JZ(-3+Z^2)+4J^3(F_2^2+m'+m'^2)Z(-3+Z^2)-J^4(F_2^2+m'+m'^2)^2(-1+3Z^2)\\
&-4J^2(-1+2F_2^2+2m'(1+m'))(-1+3Z^2))\sin(3k_Fa))
\end{aligned}
$$

$$
\begin{aligned}
B(\varphi) = & -2J^2e^{6ik_Fa}(-64F_2^4J^2(1+6(Z^2+Z^4))-3(1+2m')^2(32(Z^2+Z^4)+J^2(1+6(Z^2+Z^4)))+4F_2^2(-J^2(5\\
&+4m'(1+m'))(1+6(Z^2+Z^4))-16(1+8(Z^2+Z^4)))+4J^2\cos(\varphi)+16F_2^2J^2\cos(\varphi)-64F_2^4J^2\cos(\varphi)\\
&+16J^2m'\cos(\varphi)+16J^2m'^2\cos(\varphi)+128Z^2\cos(\varphi)+512F_2^2Z^2\cos(\varphi)+24J^2Z^2\cos(\varphi)+96F_2^2J^2Z^2\cos(\varphi)\\
&-384F_2^4J^2Z^2\cos(\varphi)+512m'Z^2\cos(\varphi)+96J^2m'Z^2\cos(\varphi)+512m'^2Z^2\cos(\varphi)+96J^2m'^2Z^2\cos(\varphi)\\
&+128Z^4\cos(\varphi)+512F_2^2Z^4\cos(\varphi)+24J^2Z^4\cos(\varphi)+96F_2^2J^2Z^4\cos(\varphi)-384F_2^4J^2Z^4\cos(\varphi)\\
&+512m'Z^4\cos(\varphi)+96J^2m'Z^4\cos(\varphi)+512m'^2Z^4\cos(\varphi)+96J^2m'^2Z^4\cos(\varphi)-8JZ(1+2Z^2)\cos(k_Fa)\\
&(-8F_2^2-(1+2m')^2+(1+2m')^2\cos(\varphi))(-4+JZ+4F_2^2JZ+(4+(-1+4F_2^2)JZ)\cos(\varphi))+4Z^2\cos(2k_Fa)\\
&(-8F_2^2-(1+2m')^2+(1+2m')^2\cos(\varphi))(16-16JZ-16Z^2+(1+4F_2^2)J^2(-1+Z^2)+(16JZ+16(-1+Z^2)\\
&+(-1+4F_2^2)J^2(-1+Z^2))\cos(\varphi))+64F_2^2\cos(2\varphi)J^2\cos(2\varphi)+4F_2^2J^2\cos(2\varphi)-4J^2m'\cos(2\varphi)\\
&+16F_2^2J^2m'\cos(2\varphi)-4J^2m'^2\cos(2\varphi)+16F_2^2J^2m'^2\cos(2\varphi)-32Z^2\cos(2\varphi)-6J^2Z^2\cos(2\varphi)\\
&+24F_2^2J^2Z^2\cos(2\varphi)-128m'Z^2\cos(2\varphi)-24J^2m'Z^2\cos(2\varphi)+96F_2^2J^2m'Z^2\cos(2\varphi)-128m'^2Z^2\cos(2\varphi)\\
&-24J^2m'^2Z^2\cos(2\varphi)+96F_2^2J^2m'^2Z^2\cos(2\varphi)-32Z^4\cos(2\varphi)-6J^2Z^4\cos(2\varphi)+24F_2^2J^2Z^4\cos(2\varphi)\\
&-128m'Z^4\cos(2\varphi)-24J^2m'Z^4\cos(2\varphi)+96F_2^2J^2m'Z^4\cos(2\varphi)-128m'^2Z^4\cos(2\varphi)-24J^2m'^2Z^4\cos(2\varphi)\\
&+96F_2^2J^2m'^2Z^4\cos(2\varphi)+8JZ(1+2Z^2)(-8F_2^2-(1+2m')^2+(1+2m')^2\cos(\varphi))(J+4F_2^2J+4Z+(-J\\
&+4F_2^2J-4Z)\cos(\varphi))\sin(k_Fa)-8Z^2(-8F_2^2-(1+2m')^2+(1+2m')^2\cos(\varphi))(-16Z+J(-4+Z(J+4F_2^2J+4Z))\\
&+(16Z+J(4+(-1+4F_2^2)JZ-4Z^2))\cos(\varphi))\sin(2k_Fa))
\end{aligned}
$$

$$
\begin{aligned}
C = & e^{3ik_Fa}(-1-2Z^2+2Z^2\cos(k_Fa)-2Z\sin(k_Fa))(4J^2(1+2F_2^2+2m'(1+m')+2Z^2-4(F_2^2+m'+m'^2)Z^2-2(-1+2F_2^2\\
&+2m'(1+m'))Z^4)+16(1+6(Z^2+Z^4))+J^4(F_2^2+m'+m'^2)^2(1+6(Z^2+Z^4))+2Z(-2(-4+J^2(F_2^2+m'+m'^2))\\
&(1+2Z^2)(4Z+J(2+J(F_2^2+m'+m'^2)Z))\cos(k_Fa)+Z(4(-1+Z)+J(J(F_2^2+m'+m'^2)(-1+Z)+2(1+Z)))\\
&(4(1+Z)+J(2-2Z+J(F_2^2+m'+m'^2)(1+Z)))\cos(2k_Fa)+2(-4+J^2(F_2^2+m'+m'^2))(4+J(J(F_2^2+m'+m'^2)\\
&-2Z))(1+2Z^2)\sin(k_Fa)+2Z(-4+J(-J(F_2^2+m'+m'^2)+2Z))(4Z+J(2+J(F_2^2+m'+m'^2)Z))\sin(2k_Fa)))
\end{aligned}
$$

IV. TABLE

We study the effect of high spin states of HSM on the Josephson supercurrent (Eq. (8)) in section 11 of our paper. Herein below we provide the spin flip probability (F_2) values of the HSM for different S and m' in a tabular format.

TABLE I: Spin flip probability (F_2) values of the HSM for different S and m'

S	m'	F_2
$\frac{1}{2}$	$-\frac{1}{2}$	1
	$\frac{1}{2}$	0
$\frac{3}{2}$	$-\frac{3}{2}$	$\sqrt{3}$
	$-\frac{1}{2}$	2
	$\frac{1}{2}$	$\sqrt{3}$
	$\frac{3}{2}$	0
$\frac{5}{2}$	$-\frac{5}{2}$	$\sqrt{5}$
	$-\frac{3}{2}$	$2\sqrt{2}$
	$-\frac{1}{2}$	3
	$\frac{1}{2}$	$2\sqrt{2}$
	$\frac{3}{2}$	$\sqrt{5}$
	$\frac{5}{2}$	0
$\frac{7}{2}$	$-\frac{7}{2}$	$\sqrt{7}$
	$-\frac{5}{2}$	$2\sqrt{3}$
	$-\frac{3}{2}$	$\sqrt{15}$
	$-\frac{1}{2}$	4
	$\frac{1}{2}$	$\sqrt{15}$
	$\frac{3}{2}$	$2\sqrt{3}$
	$\frac{5}{2}$	$\sqrt{7}$
	$\frac{7}{2}$	0
$\frac{9}{2}$	$-\frac{9}{2}$	3
	$-\frac{7}{2}$	4
	$-\frac{5}{2}$	$\sqrt{21}$
	$-\frac{3}{2}$	$2\sqrt{6}$
	$-\frac{1}{2}$	5
	$\frac{1}{2}$	$2\sqrt{6}$
	$\frac{3}{2}$	$\sqrt{21}$
	$\frac{5}{2}$	4
	$\frac{7}{2}$	3
	$\frac{9}{2}$	0
$\frac{11}{2}$	$-\frac{11}{2}$	$\sqrt{11}$
	$-\frac{9}{2}$	$2\sqrt{5}$
	$-\frac{7}{2}$	$3\sqrt{3}$
	$-\frac{5}{2}$	$4\sqrt{2}$
	$-\frac{3}{2}$	$\sqrt{35}$
	$-\frac{1}{2}$	6
	$\frac{1}{2}$	$\sqrt{35}$
	$\frac{3}{2}$	$4\sqrt{2}$
	$\frac{5}{2}$	$3\sqrt{3}$
	$\frac{7}{2}$	$2\sqrt{5}$
	$\frac{9}{2}$	$\sqrt{11}$
	$\frac{11}{2}$	0
$\frac{13}{2}$	$-\frac{13}{2}$	$\sqrt{13}$
	$-\frac{11}{2}$	$2\sqrt{6}$
	$-\frac{9}{2}$	$\sqrt{33}$
	$-\frac{7}{2}$	$2\sqrt{10}$
	$-\frac{5}{2}$	$3\sqrt{5}$
	$-\frac{3}{2}$	$4\sqrt{3}$
	$-\frac{1}{2}$	7
	$\frac{1}{2}$	$4\sqrt{3}$
	$\frac{3}{2}$	$3\sqrt{5}$
	$\frac{5}{2}$	$2\sqrt{10}$
	$\frac{7}{2}$	$\sqrt{33}$
	$\frac{9}{2}$	$2\sqrt{6}$
	$\frac{11}{2}$	$\sqrt{13}$
	$\frac{13}{2}$	0
$\frac{15}{2}$	$-\frac{15}{2}$	$\sqrt{15}$
	$-\frac{13}{2}$	$2\sqrt{7}$
	$-\frac{11}{2}$	$\sqrt{39}$
	$-\frac{9}{2}$	$4\sqrt{3}$
	$-\frac{7}{2}$	$\sqrt{55}$
	$-\frac{5}{2}$	$2\sqrt{15}$
	$-\frac{3}{2}$	$3\sqrt{7}$
	$-\frac{1}{2}$	8
	$\frac{1}{2}$	$3\sqrt{7}$
	$\frac{3}{2}$	$2\sqrt{15}$
	$\frac{5}{2}$	$\sqrt{55}$
	$\frac{7}{2}$	$4\sqrt{3}$
	$\frac{9}{2}$	$\sqrt{39}$
	$\frac{11}{2}$	$2\sqrt{7}$
	$\frac{13}{2}$	$\sqrt{15}$
	$\frac{15}{2}$	0
$\frac{17}{2}$	$-\frac{17}{2}$	$\sqrt{17}$
	$-\frac{15}{2}$	$4\sqrt{2}$
	$-\frac{13}{2}$	$3\sqrt{5}$
	$-\frac{11}{2}$	$2\sqrt{14}$
	$-\frac{9}{2}$	$\sqrt{65}$
	$-\frac{7}{2}$	$6\sqrt{2}$
	$-\frac{5}{2}$	$\sqrt{77}$
	$-\frac{3}{2}$	$4\sqrt{5}$
	$-\frac{1}{2}$	9
	$\frac{1}{2}$	$4\sqrt{5}$
	$\frac{3}{2}$	$\sqrt{77}$
	$\frac{5}{2}$	$6\sqrt{2}$
	$\frac{7}{2}$	$\sqrt{65}$
	$\frac{9}{2}$	$2\sqrt{14}$
	$\frac{11}{2}$	$3\sqrt{5}$
	$\frac{13}{2}$	$4\sqrt{2}$
	$\frac{15}{2}$	$\sqrt{17}$
	$\frac{17}{2}$	0
$\frac{19}{2}$	$-\frac{19}{2}$	$\sqrt{19}$
	$-\frac{17}{2}$	6
	$-\frac{15}{2}$	$\sqrt{51}$
	$-\frac{13}{2}$	8
	$-\frac{11}{2}$	$5\sqrt{3}$
	$-\frac{9}{2}$	$2\sqrt{21}$
	$-\frac{7}{2}$	$\sqrt{91}$
	$-\frac{5}{2}$	$4\sqrt{6}$
	$-\frac{3}{2}$	$3\sqrt{11}$
	$-\frac{1}{2}$	10
	$\frac{1}{2}$	$3\sqrt{11}$
	$\frac{3}{2}$	$4\sqrt{6}$
	$\frac{5}{2}$	$\sqrt{91}$
	$\frac{7}{2}$	$2\sqrt{21}$
	$\frac{9}{2}$	$5\sqrt{3}$
	$\frac{11}{2}$	8
	$\frac{13}{2}$	$\sqrt{51}$
	$\frac{15}{2}$	6
	$\frac{17}{2}$	$\sqrt{19}$
	$\frac{19}{2}$	0

* colin.nano@gmail.com

[1] Enoksen, H., Linder, J. & Sudbø, A. Spin-flip scattering and critical currents in ballistic half-metallic d-wave Josephson junctions. Phys. Rev. B 85,014512 (2012).

[2] Krichevsky, A., Schechter, M., Imry, Y. & Levinson, Y. Spectrum and thermodynamic currents in one-dimensional Josephson elements. Phys. Rev. B 61, 3723 (2000).

[3] de Menezes, O. L. T. & Helman, J. S. Spin flip enhancement at resonant transmission. American Journal of Physics 53, 1100 (1985).
